# The Role of LEF1 in Endometrial Gland Formation and Carcinogenesis

**DOI:** 10.1371/journal.pone.0040312

**Published:** 2012-07-06

**Authors:** Dawne N. Shelton, Hubert Fornalik, Traci Neff, Soo Yeun Park, David Bender, Koen DeGeest, Xiaoming Liu, Weiliang Xie, David K. Meyerholz, John F. Engelhardt, Michael J. Goodheart

**Affiliations:** 1 Department of Obstetrics and Gynecology, Holden Comprehensive Cancer Center, The University of Iowa Hospitals and Clinics, Iowa City, Iowa, United States of America; 2 Department of Anatomy and Cell Biology, The University of Iowa Carver College of Medicine, Iowa City, Iowa, United States of America; 3 Department of Pathology, The University of Iowa Hospitals and Clinics, Iowa City, Iowa, United States of America; The Moffitt Cancer Center & Research Institute, United States of America

## Abstract

Endometrial carcinoma is the most common gynecologic cancer, yet the mechanisms underlying this disease process are poorly understood. We hypothesized that Lef1 is required for endometrial gland formation within the uterus and is overexpressed in endometrial cancer. Using Lef1 knockout (KO) mice, we compared uterine gland development to wild-type (WT) controls, with respect to both morphology and expression of the Lef1 targets, cyclin D1 and MMP7. We characterized the dynamics of Lef1 protein expression during gland development and the mouse estrus cycle, by immunostaining and Western blot. Finally, we investigated the roles of cyclin D1 and MMP7 in gland and cancer formation in the mouse, and assessed the relevance of Lef1 to human cancer by comparing expression levels in cancerous and normal endometrial tissues. Lef1 upregulation in mouse endometrium correlates with the proliferative stages of the estrus cycle and gland development during the neonatal period. WT mice endometrial glands began to develop by day 5 and were easily identified by day 9, whereas Lef1 KO mice endometrial glands had not developed by day 9 although the endometrial lining was intact. We found that during gland development cyclin D1 is elevated and localized to the gland buds, and that this requires the presence of Lef1. We also noted that Lef1 protein was expressed at higher levels in endometrial cancers within mice and humans when compared to normal endometrium. Our loss-of-function data indicate that Lef1 is required for the formation of endometrial glands in the mouse uterus. Lef1 protein elevation corresponds to gland formation during development, and varies cyclically with the mouse estrus cycle, in parallel with gland regeneration. Finally, Lef1 is overexpressed in human and mouse endometrial tumors, consistent with it playing a role in gland proliferation.

## Introduction

Endometrial carcinoma is the most common gynecologic malignancy and ranks second as a cause of gynecologic cancer mortality in the United States; in 2012, the American Cancer Society predicts 47,130 new cases and 8,010 deaths in this year alone [1]. Most endometrial carcinomas arise from the glands of the endometrium (adenocarcinomas), and the remainder from the supporting stroma (sarcomas). Currently, we do not know which proteins regulate endometrial-gland development and proliferation, or which molecular interactions trigger cancer development within the uterus. Clearly, the mechanisms that regulate cyclic turnover of the endometrium, as well as those that control continual regeneration of the glands and supporting stroma during this turnover, are precisely regulated. In other mammals, such as mice, endometrial turnover takes as few as 4 days, but in humans it can last as long as 30 days. Identifying the molecular pathways that govern cyclic turnover of the endometrium and the factors that initiate gland development will help us understand the regulatory defects that give rise to cancer. Additionally, a more comprehensive understanding of endometrial gland formation will provide insight into: 1) other disorders involving gland dysregulation (e.g., endometriosis, infertility, menstrual irregularities and adenomyosis), and 2) disorders involving hyperproliferation of cells within the endometrial glands, for example complex hyperplasia (with and without atypia) and cancer.

Uterine adenogenesis refers to the formation of glands within the uterus. This process in mammals is mainly a postnatal event, and involves: 1) differentiation and budding of the glandular epithelium, 2) invagination and tubular coiling of the epithelium and, 3) branching of the glandular elements and their expansion throughout the endometrial stroma toward the myometrium [2]. Wild-type (WT) mice lack endometrial glands at birth, but exhibit epithelial invaginations, or gland buds, by postnatal day (PND) 5. The beginnings of endometrial glands are present by PND 7, and complex glands (extending from the endometrial lumen through the stroma) are observed by PND 10 [3], [4]. In humans, endometrial gland development begins *in utero*, with superficial endometrial glands present by 20–22 weeks of gestation. Endometrial glands continue to develop from birth to age 6, reaching half the distance to the myometrium by the end of this period. They are mature and fully developed by puberty. In human females, the menstrual cycle takes approximately 28 days to complete, and is comprised of two phases: the follicular or growth phase (proestrus/estrus in the mouse) and the luteal or regression phase (metaestrus/diestrus in the mouse). The human endometrium can easily be dated by endometrial biopsy, and can be classified with respect to the different phases or days of the menstrual cycle. Although mice do not menstruate, they likewise have biochemically and histologically distinct phases of estrus [5], [6].

Lymphoid Enhancing Factor 1 (Lef1), a member of the TCF/LEF family, is a 44.2 kDa protein comprised of 399 amino acids. During Wnt signaling, the transcriptional activity of Lef1 is upregulated as a consequence of the translocation of β-catenin from the cytoplasm to the nucleus; in this context, Lef1 and other TCF factors transcriptionally up-regulate multiple downstream target genes [7] – including cyclin D1, MMP7 and c-myc [8], [9], [10], [11]. Notably, c-myc activity has been implicated in the etiology of endometrial carcinomas, and cyclin D1 is upregulated in endometrial carcinomas [12], [13], [14]. LEF1 itself is essential, in a variety of tissues, for the development of structures that require interactions between epithelia and mesenchyme; for example, Lef1 KO mice lack airway submucosal glands, hair follicles and mammary glands [15], [16]. Wnt-pathway signaling is also relevant in the uterus. For example, both Wnt7a (expressed in the luminal epithelium) and Wnt5a (expressed in the mesenchyme) are required for epithelial mesenchymal interactions and gland development in this organ. Mutations of the CTNNB1 gene, which commonly occur in 12–25% of endometrial carcinomas, typically result in increased cytoplasmic and nuclear accumulation of β-catenin, which subsequently leads to activation of Lef1 and Tcf family members. This relationship between mutated β-catenin leading to activated Lef1 gave us cause to speculate that Lef1 may be dysregulated in endometrial cancer [17], [18], [19]. Overall, this literature suggests that the Wnt/β-catenin/Lef1 pathway may contribute to both the regulation of uterine growth (at least in the context of cancer), and normal development and function of the uterus [20], [21], [22]. We hypothesized that Lef1 is required for endometrial gland formation within the uterus and is overexpressed in endometrial cancer. Through the experiments below we tested the roles of Lef1 in normal development of the endometrial gland, as well as in the emergence of cancer.

## Materials and Methods

### Lef1 Knockout Mice and Wild Type Controls

Lef1 knockout mice were generated by inserting a targeting gene construct using a phosphoglycerokinase (PGK)-neo cassette into the second exon of the HMG domain of Lef1 as previously described [16]. These mice are easily identified as they posses a characteristic pointed snout and lack vibrissae. Since the majority (95%) of the original Lef1 mice die within the first two weeks of life we attempted to try and increase their survival by breeding the original C57BL\6 onto an ICR background. The mice used for these experiments were Lef1 C57B6/ICR F1 mice. This strain was obtained via our collaborative relationship with JFE. Wild type female ICR mice were obtained from Harlan Laboratories (Indianapolis, IN). All mice were housed under pathogen free conditions and a 12-hour dark/light cycle. Mice were given food and water *ad libitum*. All animal experiments conformed to NIH guidelines for the care and use of animals and were approved by the University of Iowa Institutional Animal Care and Use Committee (Approval # 1011224).

### Mouse Estrus Cycle

Female ICR mice aged 8–10 weeks, obtained from Harlan Laboratories (Indianapolis, IN), were housed together, and estrus cycling was initiated by exposure of the female mice to the dirty bedding of male mice, according to the technique described by Whitten [23]. Starting 72 hours after this exposure, mice were sacrificed daily over the course of five days. One hour prior to sacrifice, each animal was injected (intraperitoneal) with 400 µl of 5 mg/ml BrdU. Ovaries, uterine horns, cervix and vagina were taken as samples. For each animal, one uterine horn/ovary and the vagina were embedded into paraffin and cut into 4 µm sections; the other uterine horn/ovary was embedded into Optimal Cutting Temperature (OCT) compound and cut into 10 µm sections. Vaginal swabs were also obtained (using saline-moistened, cotton-tipped applicators) and cytologically stained. The phase of the estrus cycle was assigned by a veterinarian pathologist (DKM) based on vaginal cytology, vaginal histology and uterine histology.

### Lef1 Protein Expression during Endometrial Development and the Estrus Cycle

Western Blot analysis of protein lysates was performed on both the developing uterus (PND 3, 5, 7, and 9) and cycling uterus (phases: diestrus, proestrus, estrus, metaestrus). Lysates were run on a 10% SDS page gel with molecular weight markers, for 2.5 hours at 80 volts, and then transferred to nitrocellulose paper for one hour at 400 milliamps. The nitrocellulose was then incubated in blocking buffer (0.1% casein and 0.2% Tween 20). Blots were probed for Lef1 using anti-Lef1 antibody (Exalpha Biologicals T100M) at a dilution of 1∶1,000, and for GAPDH using goat polyclonal anti-GAPDH (Abcam ab9483) at 1∶1,000. In the case of the development experiments, samples representing a single time point were pooled (approximately 10–15 uterine horns per PND) to generate sufficient protein for the analysis, and the experiments were repeated 3 times. In the case of the estrus-cycle experiments, however, pooling was not necessary because each adult uterine horn yielded an adequate amount of protein. Each experiment was repeated 3 times per cycle time point. For each Western Blot, protein was quantified by the Bradford method, and equal amounts of protein were loaded onto each gel. Quantification was performed using Image J software [24].

### Paraffin Embedding and Hematoxylin and Eosin (H&E) Staining

The uterine horn was removed from Lef1 KO and ICR WT control mice on PND 3, 5, 7 and 9. The mouse uterus was fixed in 10% neutral buffered formalin overnight, processed and then embedded into paraffin. Next, 4 µm sections were cut and H&E staining was performed. Photomicrographs were taken at 20× and 40× magnification.

### Cyclin D1 Protein Expression/BrdU Incorporation

Staining for cyclin D1 expression and BrdU incorporation was performed on both developing and cycling uteri. Briefly, OCT-embedded sections were cut (10 µm), fixed and stained using mouse anti-BrdU antibody 1∶200 (Roche 11170376001) overnight at 4°C, or anti-cyclin D1 antibody diluted by the vendor (Abcam ab15196) for 1 hour at room temperature. Slides were washed twice for 2 minutes in PBS, and a FITC-conjugated donkey anti-rabbit secondary antibody (Jackson Labs 711-096-152) was applied for 1 hr at room temperature to detect the cyclin D1 primary antibody. Slides were then washed twice for 2 minutes and incubated with Texas Red-conjugated donkey anti-mouse antibody (Jackson Labs 715-076-150) at room temperature for 1 hour to detect the BrdU primary antibody. Finally, slides were washed twice for 5 minutes, and then cover slipped with Vectashield containing DAPI (Vector Labs H-1200) for detection of nuclei. Collages of photomicrographs were generated using Adobe Photoshop CS3, and signal (cyclin D1, BrdU, and DAPI) within both the glandular elements and the endometrial luminal epithelium were quantified using MetaMorph software version 7.5 (MDS Analytical Technologies, Downingtown, PA). With regard to cyclin D1 staining, only nuclear staining was counted as positive, and expression was evaluated as the number of glandular cyclin D1-positive nuclei/total glandular (DAPI-positive) nuclei. These experiments were carried out in triplicate, and the values were averaged and plotted against either PND of development or phase of the estrus cycle.

### Collection of Human Uterine Tissue (Normal and Cancerous)

After obtaining approval for our protocols from the University of Iowa Institutional Review Board, and informed written consent from patients, we collected endometrial tissue from uteri removed during hysterectomies performed at the University of Iowa Hospitals and Clinics (UIHC). Samples were prepared within 15–30 minutes of surgery. A portion of the sample was snap frozen in liquid nitrogen. The other portion was transported to the laboratory in PBS and subsequently embedded in OCT. These samples included transverse sections that were cut from portions of the uterus that contained grossly cancerous tissues, and included some of the normal underlying myometrium. Both snap-frozen and embedded tissue were stored at −80°C. All surgical specimens were histologically assessed by UIHC staff pathologists, for confirmation of diagnosis and review during weekly Gynecologic Oncology Tumor Board meetings.

### Collection of Mouse Endometrial-cancer Samples

Endometrial cancer was induced in ICR mice, by anaesthetizing them with ketamine/xylazine and injecting 1 mg/100 g (mouse) of N-methyl-N-nitrosourea (NMU) into the left and right uterine corpora. Mice were then fed a diet including 5 ppm 17β-estradiol (E2) for six months, as described previously [25]. Harvested uteri were snap frozen in liquid nitrogen and embedded in OCT, and a separate sample of uterine tissue was preserved for evaluation by H&E. All slides were reviewed by a veterinarian pathologist (DKM) to determine whether cancerous tissue was present.

### Immunofluorescence of Lef1 Staining Cells

All slides for Lef1 staining were embedded in OCT, cut (10 µm), fixed in 4% paraformaldehyde and washed three times for 5 minutes. After blocking for 1 hour with donkey serum, slides were washed in PBS and then stained with Lef1 antibody (Cell Signaling Technology, Cat. #2230) at 1∶1000 dilution overnight at 4°C for post-natal sections or 1∶200 dilution for 1 hour at room temperature for all other tissues. Following washing, FITC-conjugated donkey anti-rabbit secondary antibody (Jackson Labs 711-096-152) was applied at 1∶250 dilution. Samples were washed twice for 5 minutes each and then cover slipped with Vectashield containing DAPI (Vector Labs H-1200) for detection of nuclei.

### RNA Extraction

Snap-frozen tissue was either immediately pulverized or stored in RNA*later®*-ICE Solution (Applied Biosystems/Ambion, Austin, TX). Samples were then homogenized using a FP120 FastPrep machine (Thermo Savant, Asheville, NC) and RNA was extracted using either the mirVana™ miRNA isolation Kit (Applied Biosystems/Ambion, Austin, TX) or RNeasy® Mini Kit (Qiagen, Valencia, CA), according to the manufacturer’s instructions. RNA quality was assessed by running samples on the RNA 6000 Nano chip according to manufacturer’s specifications, and RNA integrity numbers (RIN) were generated using Agilent 2100 Expert Software v B.02.07 (Agilent Technologies, Palo Alto, CA). Specimens with RIN numbers equal to or greater than 5 were considered to be of acceptable quality, and were included in further analysis.

### Quantitative PCR

Single-stranded cDNA was synthesized from 2 µg of total RNA using the Superscript III kit (Invitrogen, Carlsbad, CA). PCR was quantitated using the BioRad CFX96 quantitative Real-Time PCR detection system and software, and the iQ SYBR Green SuperMix (BioRad, Hercules, CA). All samples were run in duplicate and cDNA levels were normalized to those for ß-actin. The sequences of the PCR primers used were as follows: h.s.Lef1-F 5′-cgaagaggaaggcgatttag-3′; h.s.Lef1-R 5′-gagaggtttgtgcttgtctgg-3′; h.s.cyclin D1-F 5′-gccctcggtgtcctacttc-3′; h.s.cyclin D1-R 5′-aagacctcctcctcgcactt-3′; h.s.MMP7-F 5′-agttgtatggggaactgctga-3′; h.s.MMP7-R 5′-agactgctaccatccgtcca-3′; h.s.ß-actin-F 5′-acagagcctcgcctttgccg-3′; h.s.ß-actin-R 5′-acccatgcccaccatcacgc-3′. The amplification conditions were: 10 seconds of denaturation at 95°C, 10 seconds of annealing at 60°C, and 20 seconds of extension at 72°C, for a total of 40 cycles. A template-free negative control was included in each experiment. Fold differences in RNA levels were adjusted for weight and height as measured by body mass index (BMI) and age. Statistical analyses were performed using GraphPad Prism (version 5.0b).

## Results

### Lef1 Protein Expression in the Newborn Mouse Uterus Correlates with Timing of Uterine-gland Formation

**Figure 1:**
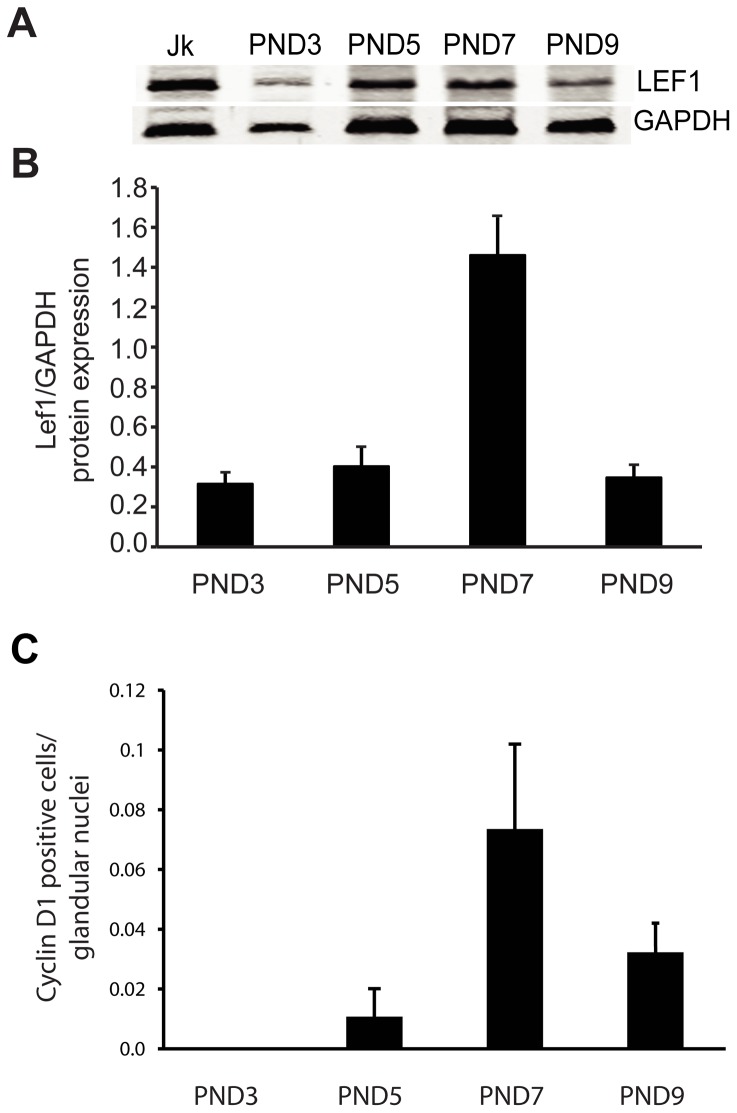
Lef1 is expressed in the developing mouse uterus. (A) Western blot analysis assessing Lef1 expression in uterine samples taken from mice on post-natal days (PNDs) 3, 5, 7, and 9. For each sample, mouse uteri from multiple animals were pooled and used to generate lysates; equal amounts of protein were loaded. Jurkatt (Jk) cells were used as a positive control, and GAPDH was used as a loading control. (B) Quantitative analysis of Lef1 protein expression, as measured by Image-J analysis and plotted against developmental day. Relative Lef1 protein expression increased between PNDs 5 and 7, coinciding with the period during which mouse uterine glands become histologically identifiable. (C) Quantitative analysis of cyclin D1 expression in the glandular epithelium, as assessed by IF analysis of developing mouse uteri (the number of cells positive for cyclin D1 expression was divided by the total number of nuclei within the glandular epithelium). Error bars represent SEM.

We hypothesized that the developmental programs responsible for endometrial-gland proliferation may be similar to those that are dysregulated in endometrial cancers. We tested this by examining Lef1 expression during uterine development. We first evaluated Lef1 expression within the uteri of newborn ICR mice, on PNDs 3, 5, 7, and 9 (Fig. 1). Due to the small size of the uterus at this time, 10–15 uterine horns per sample were pooled for western blotting (Fig. 1A). Lef1 protein levels gradually increased, peaking on PND 7; the levels declined to nearly starting levels by PND 9 (Fig. 1B). Uterine-gland buds are first visible histologically on or around PND 5, depending on the mouse species and strain. They subsequently undergo maturation and coiling, and then invaginate into the underlying stroma. Adenogenesis continues until PNDs 7–9, and then tapers off until the animals reach sexual maturity (approximately 6 weeks), at which time estrus cycling begins. Notably, this decrease in the number of gland buds coincides with the decrease in Lef1 protein levels starting after PND 7. On PND 9, when the majority of gland formation is complete, Lef1 protein levels are back to near-baseline levels.

We next examined expression of the Lef1 target cyclin D1 in the developing mouse uterus because cyclin D1 levels are elevated in patients with endometrial carcinoma [8], [12], [13]. Not only elevations in cyclin D1 protein, but also mutations that lead to such increases, have been reported in patients with endometrial cancer [26]. Given that these increases in cyclin D1 levels can lead to more rapid entry into the cell cycle and to increased cell proliferation within target tissues, cyclin D1 is considered an oncogene [27]. Similar to Lef1, cyclin D1 is expressed in the developing gland buds, with levels peaking at PND 7 (the time of maximal gland formation) and then decreasing significantly by PND 9 (when gland formation is complete; Fig. 1C).

### During the Mouse Estrus Cycle, Lef1 Expression Peaks During the Phase of High Cell Proliferation within the Glands

**Figure 2:**
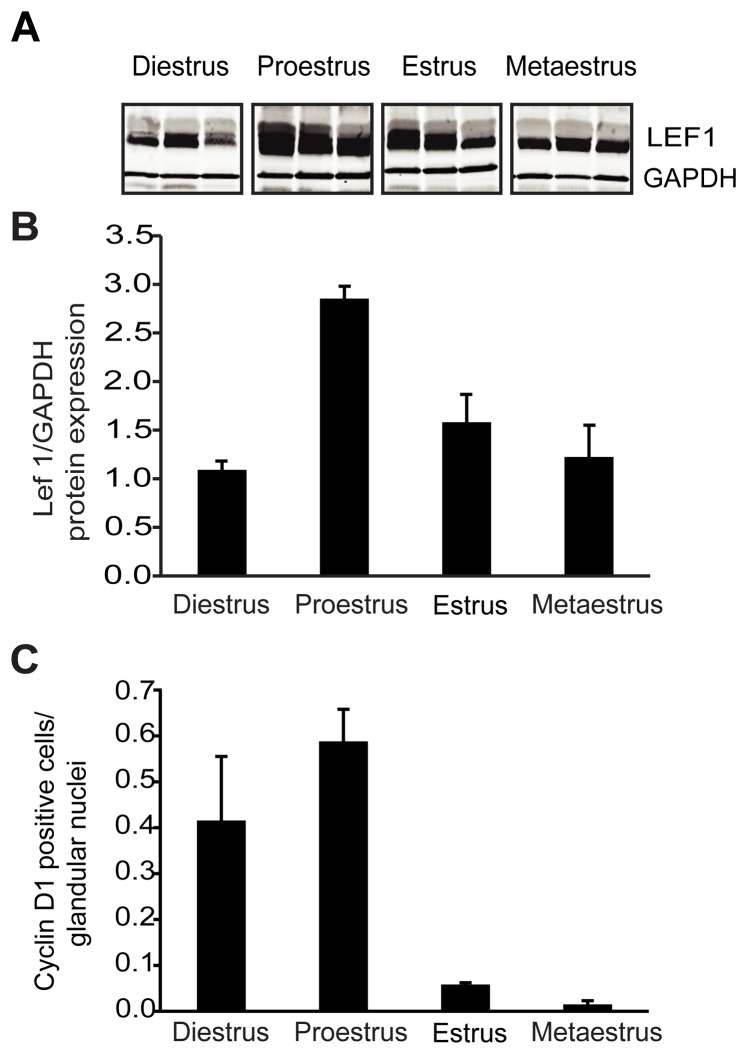
Lef1 expression changes over the course of the mouse estrus cycle. (A) Mouse uteri were segregated based on phase of the estrus cycle (n = 3 per phase) and evaluated for Lef1 protein expression by Western blotting; GAPDH was used as a loading control. (B) Quantitative analysis of Lef1 protein expression (as measured by Image-J analysis) according to phase of the estrus cycle (average of values for all samples in a given phase, with error bars). Note the increase in Lef1 protein levels during proestrus, the time at which gland development is at its peak. (C) Quantitative analysis of cyclin D1 expression in the glandular epithelium (the number of cells positive for cyclin D1 was divided by the total number of nuclei within the glandular epithelium). Note that cyclin D1 levels peak during proestrus, coinciding with the peak in gland development and the initial increase in Lef1 levels. Error bars represent SEM.

We next examined Lef1 expression in adult ICR mouse uteri over the course of the estrus cycle (Fig. 2). Like uteri in humans, those of the mouse respond cyclically to circulating hormones produced during ovulation. Cell proliferation commences during diestrus, peaks at proestrus, begins to recede during estrus, and stops during metaestrus. Western blot analysis of protein lysates prepared from staged uteri revealed that Lef1 expression is maximal during proestrus, and also high during estrus (Fig. 2A,B). The elevation in Lef1 protein correlates with the period of maximal cellular proliferation and gland formation, i.e. proestrus, as previously established by the quantification of levels of proliferating cell nuclear antigen (PCNA) [28]. We also observed that levels of cyclin D1 expression were high during proestrus (Fig. 2C), as expected given that cyclin D1 is a downstream target of Lef1 and regulates cell proliferation.

### Lef1 Knockout Mice do not Develop Endometrial Glands Despite having a Normal Endometrial Lining

**Figure 3:**
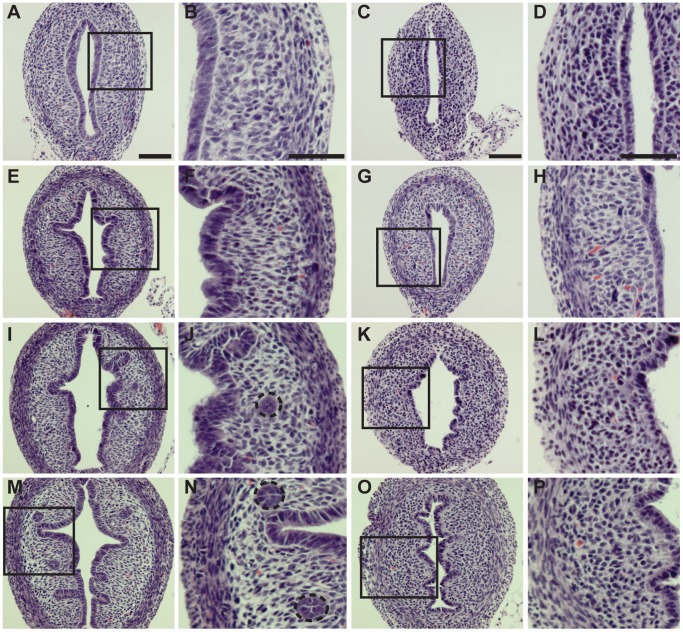
Lef1 knockout mice do not develop endometrial glands. (Two vertical columns left) H&E photomicrographs of uteri from wild-type ICR mice on post-natal days (PNDs) 3, 5, 7, and 9. Wild-type uterus at PNDs: (A, B) 3, (E, F) 5, (I, J) 7, and (M, N) 9; magifications for each PND are 200× and 400×, respectively. Note that endometrial glands are visible on PNDs 7 and 9 (dashed circles). (Two vertical columns right) H&E photomicrographs of uteri from Lef-1 knockout mice on PNDs 3, 5, 7 and 9, demonstrating that endometrial glands fail to form during this period. Lef1 knockout uterus on PNDs: (C, D) 3, (G, H) 5, (K, L) 7, and (O, P) 9; magnifications for each PND are 200× and 400× respectively.

Having observed Lef1 and cyclin D1 expression in WT mouse gland buds and glands, we decided to examine gland formation within the Lef1 KO mouse previously characterized by van Genderen et al. [16]. We compared uterine gland development in WT mice to that in Lef1 KO mice, i.e. examining mice from PND 3–9 (Fig. 3A–P). Although no glandular elements were observed on PND 3 in either WT or Lef1 KO animals, the endometrial lining was intact (Fig. 3A&B and C&D, respectively). In WT animals, gland buds were visible by PND 5 (Fig. 3E&F), glands by PND 7 (Fig. 3I&J), and glands that are complex and coiled by PND 9 (Fig. 3M&N). In the Lef1 KO mouse uterus, by contrast, endometrial glands did not develop. Although an intact endometrial lining was present within the Lef1 KO mouse uterus at PND 3 (Fig. 3C&D), gland buds failed to form by PND 5 (Fig. 3G&H), and even by PND 7 or 9 no signs of gland formation were visible (Fig. 3K&L; 3O&P). Other studies have demonstrated that glands fail to form in the absence of either Wnt7a or Wnt5a [20], [21]. Our data are consistent with Lef1 acting downstream of Wnt7a and Wnt5a within the uterus, and suggest that Lef1 expression is essential for endometrial gland development.

### Lef1 and Cyclin D1 are Expressed in the Developing Gland Buds of Wild-type Mice, but Absent from those of Lef1 Knockout Counterparts

**Figure 4:**
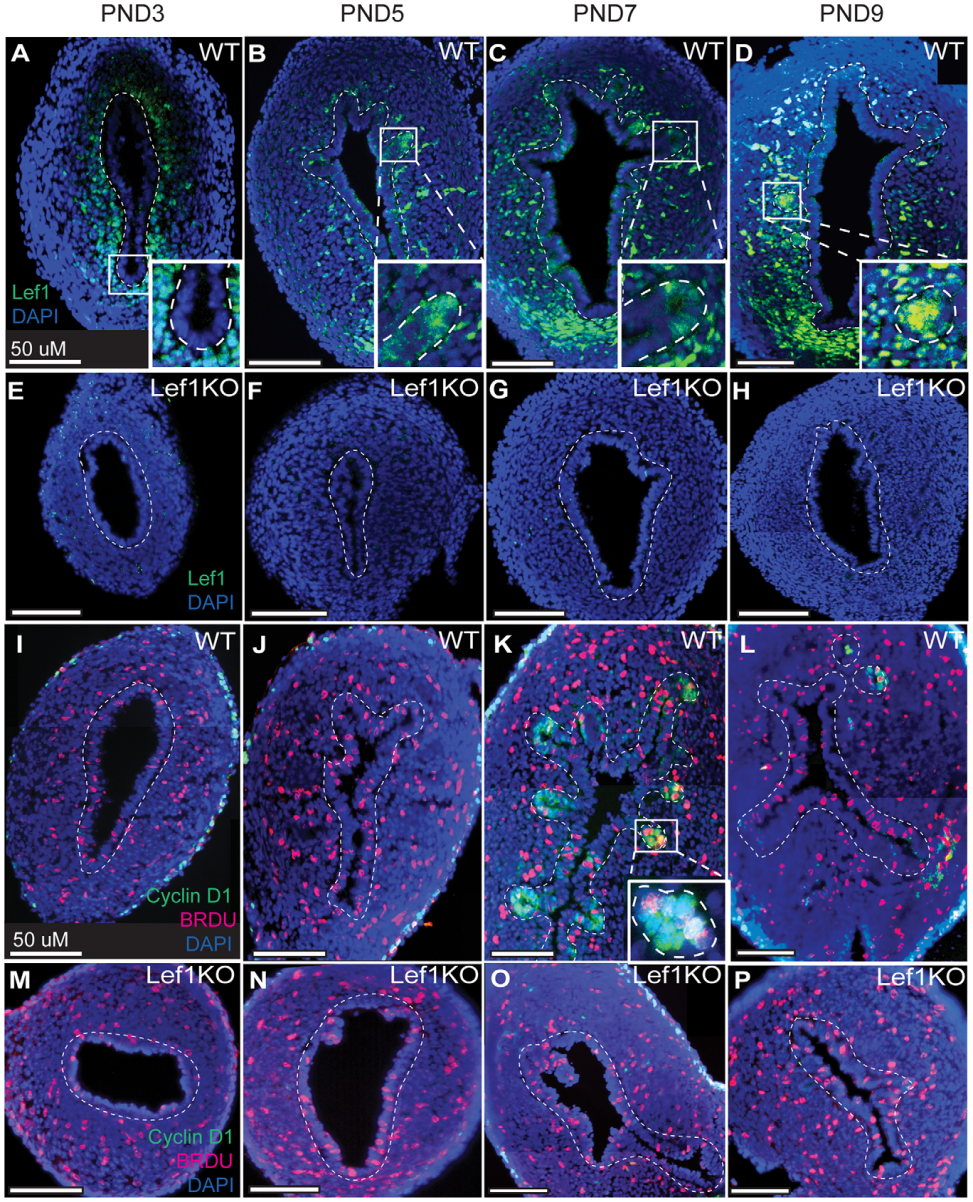
Lef1 and cyclin D1 are expressed during gland development in WT but not Lef1 KO mouse uteri. (A–P) Lef1 and/or cyclin D1/BrdU expression in the developing uterus on post-natal days (PNDs) 3 (A, E, I, M), 5 (B, F, J, N), 7 (C, G, K, O) and 9 (D, H, L, P). (A–D) Photomicrographs of Lef1 expression in wild-type uteri. Expression is present in both mesenchymal and epithelial tissue throughout this period, and is visible in the glands as they begin to form on PND 5 (panel B and inset). (E–H) Photomicrographs of Lef1 expression in Lef1 KO mice. In addition to lacking Lef1 expression, these uteri fail to form endometrial glands. (I–L) Photomicrographs of cyclin D1 expression and BrdU incorporation in wild-type uteri. Both are visible in developing endometrial glands beginning on PND 7 (panel K and inset). (M–P) Photomicrographs of cyclin D1 expression and BrDU incorporation in the uteri of Lef1 KO mice. In addition to an absence of formed glands, these uteri lack cyclin D1 expression despite the fact that cell proliferation is active, as indicated by BrdU incorporation. Dashed lines mark the border between the mesenchymal and epithelial cell layers.

Multiple studies have shown that Lef1 is essential for the inductive epithelial-mesenchymal interactions that lead to adenogenesis during development of both the trachea and mammary glands [16], [29], [30]. We assessed Lef1 expression in the developing uterus by immunofluorescent analysis. In WT mice, we observed expression in mesenchymal tissue on PNDs 3, 5, 7, and 9, and in epithelial tissue (specifically within the gland bud) beginning on PND 5 (Fig. 4A–D). As expected, in the Lef1 KO uteri, no expression was detected (Fig. 4E–H). Analysis of cyclin D1 immunostaining in WT mice revealed expression in the developing gland buds and glands at PND 7, and a reduction to near background levels by PND 9. In uteri from the Lef1 KO mice, cyclin D1 expression was completely absent (Fig. 4M–P). This loss of cyclin D1 expression was not the consequence of a lack of cell proliferation within the uterus; for all time points, BrdU injected 1 hour prior to harvest was incorporated by cells throughout the uterus (Fig. 4I–P).

### Lef1 is Overexpressed in Mouse Endometrial Cancers

**Figure 5:**
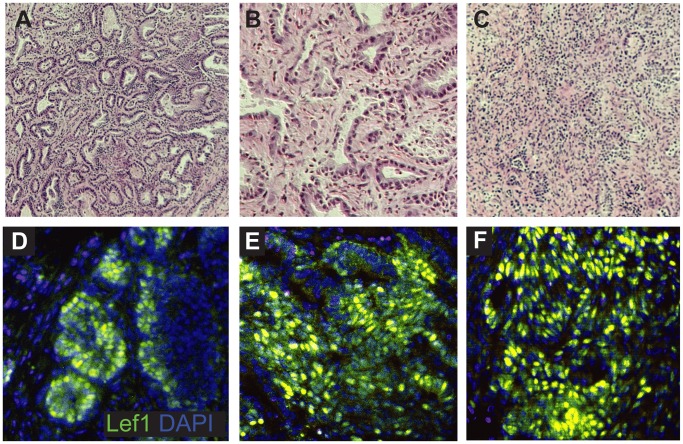
Lef1 is overexpressed in mouse endometrial cancers. (A–C) H&E photomicrographs of 3 endometrial tumors from ICR mice (wild-type strain) treated with NMU, taken at 100×, 200×, and 100× magnification, respectively. (D–F) IF images of Lef1 expression (yellow-green) in mouse endometrial tumors, shown at 200× magnification. (D–F) Note the abundance of Lef1 staining within the malignant glandular areas.

We generated endometrial tumors by injecting mouse uteri with NMU and exposing them to a diet rich in estrogen (E2) [31], [32]. We chose this model because it produces endometrial carcinomas that closely mimic Type I human endometrial cancers. The resemblance of the tumors produced in this model to grade 1 endometrial carcinomas was confirmed by histological analysis carried out by a veterinary pathologist (DKM) (Fig. 5A–C). Consistent with our findings in human endometrial cancers, Lef1 was overexpressed in the glandular component of all tumors tested (Fig. 5D–F).

### Lef1 is Overexpressed (at Protein and RNA Levels) in Human Endometrial Tumors

**Figure 6:**
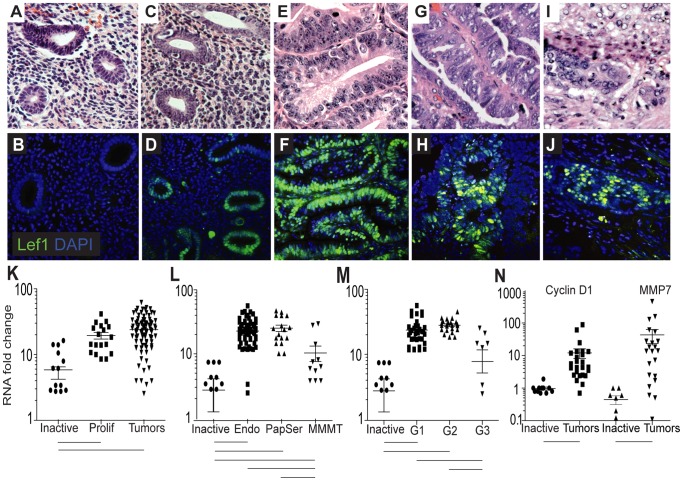
Lef1 is overexpressed in human endometrial cancers. (A–J) H&E and Lef-1/DAPI staining of inactive (A, B) and proliferating (C, D) normal endometrium, and of endometrioid (E, F), papillary serous (G, H), and malignant mixed mullerian (I, J) endometrial cancers. (K–M) Quantification of Lef1 expression, as assessed by real-time PCR, in a variety of tumors and normal tissues: (K) in 99 independent endometrial tumor samples of mixed histology, and in inactive and proliferating (Prolif) normal endometrium; (L) in endometrioid tumors (Endo), papillary serous tumors (PapSer), malignant mixed mullerian tumors (MMMT), and inactive endometrium; and (M) in tumors by grade only within endometrioid histology, and in inactive endometrium. (N) Cyclin D1 and MMP7 expression in endometrial cancers of mixed histology and in inactive endometrium. Bars below plots indicate sample pairs for which p-values <0.05. Error bars represent SEM.

To investigate the role of Lef1 in endometrial-gland formation and cancer, we first collected human tissue samples from 133 patients who ranged in age from 26 to 87 years, with a mean age of 64 years. Our controls were 33 endometrial samples from women with benign gynecological disorders that included normal pre- and post-menopausal inactive endometrium (Fig. 6A,B). This group of patients was selected because the average age at diagnosis for endometrial cancer is 60 (postmenopausal), and controls should be from patients in the same age group as well as free of endometrial cycling; in all cases, endometrial activity was confirmed to be inactive by a surgical pathologist. There was one sample from a premenopausal patient with proliferative endometrium (Fig. 6C,D) that was not included in the control samples or used in subsequent analyses, but was used to demonstrate Lef1 activity within the cycling uterus. The 99 tumors collected included: 70 endometrioid-type tumors (Fig. 6E,F), 12 papillary-serous-type endometrial tumors (Fig. 6G,H) and 17 malignant mixed mullerian tumors or carcinosarcomas (MMMT) (Fig. 6I,J). These samples covered a broad range of stages and grades.

Immunostaining of these tissues shows that Lef1 protein is expressed in normal proliferative tissue and endometrial tumors (Figs. 6D,F,H,J), but not in inactive postmenopausal tissue (Fig. 6B). Similarly, quantitative real-time PCR data demonstrate that Lef1 RNA expression is higher in the endometrium of patients with proliferative, non-cancerous disorders and in patients with endometrial cancer than in endometrium from normal postmenopausal patients (Fig. 6K). We next examined levels of Lef1 RNA expression across the range of tumor subtypes: endometrioid, papillary serous, and MMMT (Fig. 6L). We found that Lef1 expression differs significantly (p<0.05) between the three subtypes, with endometrioid and papillary serous histologies showing the greatest elevations in expression. As revealed by the MMMT photomicrograph, Lef1 expression was largely confined to the adenocarcinoma or glandular portions of the tumors, and not present in the sarcoma and stromal areas (Fig. 6J). Lef1 expression also differed significantly (p<0.05) between grade 1–2 and grade 3 endometrioid tumors. Although Lef1 expression was higher than normal inactive endometrium in grade 3 tumor samples, it was lower than that in grade 1 and 2 tumors (Fig. 6M). These data support the observation that grade 1 tumors (defined as those in which 95% of the tumor is making well-defined glands and no more than 5% of the tumor is composed of solid components) express large amounts of Lef1, and grade 3 tumors (those in which 50% of the tumor is making well-defined glands and 50% is composed of solid components) express less Lef1 than other tumors, but more than inactive endometrium [33]. Since Lef1 is a target of Wnt signaling, we examined the expression (at the RNA level) of other well-characterized Wnt targets, namely cyclin D1 and MMP7, which are also direct downstream targets of Lef1. We found that these RNAs were expressed at approximately 10- and 30-fold higher levels, respectively, in endometrial tumor samples than in inactive endometrium (Fig. 6N) [34], [35].

## Discussion

In contrast to what has been observed in colon cancer, the uterus normally expresses Lef1 protein both during its initial development and as it cycles through estrus (Fig. 1 & 2). During uterine development, Lef1 expression coincided with that of cyclin D1, a well-established target of Wnt/β-catenin/Lef1 signaling. Lef1 also appeared to be precisely regulated during the mouse estrus cycle. Specifically elevations coincided with proestrus, the period associated with cell proliferation within the gland, and during which elevations in cyclin D1 levels were also observed. As noted by Zysow et al., the patterns of Lef1 protein and E2 expression are similar, with maximal expression observed during proestrus [36]. Also, E2 has been shown to up-regulate both Lef1 and Tcf-3 within the mouse uterus [36], [37]. These data suggest that E2 may regulate Lef1, which could be of clinical interest given the importance of E2 in carcinogenesis within the endometrium.

We noted that Lef1 expression within the uterus is subject to temporal control during both development and endometrial-gland maintenance. Additionally the absence of Lef1 resulted in failed uterine-gland formation despite the presence of a normal endometrium. When the Lef1 KO mice were originally characterized by van Genderen et al., this group noted a lack of mammary glands and other structures that require epithelial-mesenchymal interactions, for example teeth and hair follicles [16]. Consistent with this finding, Hai et al. demonstrated that salivary-gland regeneration is dependent on Wnt/β-catenin signaling, and that inhibition of β-catenin signaling disrupted the development of mouse salivary glands [38]. These data indicate that the normal regulatory processes that contribute to gland formation in the uterus and salivary gland are dependent on Wnt signaling and, in the case of the uterus, specifically Lef1-mediated Wnt signaling.

We have identified the cells that express Lef1 during gland development, by staining the mouse uterus for both Lef1 and the Lef1 downstream target cyclin D1 during gland development. As shown in Figure 4, Lef1 is expressed in the stroma as early as PND 3, and then in the gland buds as they form at PND 5 and 7 and persists in the glands through PND 9. Cyclin D1 expression lags behind Lef1 expression, appearing in the developing gland invaginations on PND 7, and disappears from these invaginations by PND 9, at which time it is also present within the developing glands. Lef1 has a well-established role in the formation of both mammary and airway-submucosal glands [15], [16], and cyclin D1 has also been shown to play a role in mammary gland development [39]. Our data support an additional role for Lef1 and its activation of downstream targets of the Wnt signaling pathway, specifically cyclin D1 and MMP7, during endometrial-gland formation.

Our generation of mouse endometrial tumors via uterine NMU injections enabled us to confirm that high Lef1 expression is associated with uterine tumors, and to characterize Lef1 location in this context. Our discovery that Lef1 staining is abundant in both glandular structures and some of the supporting stroma (Fig. 5 D–F) support our original hypotheses (based on the examination of normal and cancerous human endometrium) that Lef1 function is essential for normal gland formation, and that Lef1 overexpression promotes cancer. We have observed high levels of gland-specific Lef1 expression in both the mouse model of endometrial cancer and in human endometrial tumors. Given the high degree (>95%) of identity between mouse and human Lef1 protein orthologs, we are confident that the mouse system represents a reliable *in vivo* model in which to test potential therapies that are based on regulation of the Wnt/β-catenin/Lef1 pathway for their effectiveness in slowing the growth of endometrial carcinomas.

In a paper addressing the effects of Wnt signaling on endometrial-gland growth, Mericskay et al. showed that when transplanted under the kidney capsule, the uterus from Lef1 KO mice developed endometrial glands [21], which would not be predicted based on our findings. We propose that this apparent discrepancy may be related to the transplantation model used by those authors, as other secreted Wnt ligands could potentially stimulate gland growth independent of Lef1 in that context. Such an effect seems feasible, as the importance of gland growth within the uterus to reproduction has probably resulted in multiple redundant pathways that ensure that this process takes place.

Our study is one of the first to examine the role of LEF1 in the development of endometrial glands and the expression within endometrial carcinomas. Aberrant overexpression of the Wnt-pathway target gene LEF1 has been detected in several cancers, principally: colon, leukemia, melanoma, and pancreatic [35], [40], [41], [42], [43]. Lef1 is ectopically expressed in colon cancer by the preferential targeting of a full-length (FL), ß-catenin-sensitive Lef1 isoform by activated ß-catenin and TCF transcription complexes. This Lef1-FL isoform then reinforces Wnt signaling by activating downstream genes including cyclin D1, c-myc, and MMP7. Our observations of an elevation in the expression of Lef1-FL in human endometrial tumors and of Lef1 expression in non-cancerous proliferative endometrium, but not in inactive endometrium (Fig. 6) are consistent with the findings from colon cancer. Thus we believe not only that Lef1 is important in endometrial-gland formation, but also that dysregulated Lef1 plays a role in the etiology of endometrial cancer.

Although endometrial cancer is commonly associated with nuclear accumulation of ß-catenin (e.g., 13–69% of cases of endometrioid endometrial carcinoma), only a few studies have examined the role of Wnt signaling in its etiology. Among these, one study by Fukuchi et al. assessed endometrial tumors for β-catenin mutations in exon 3, the site of regulatory phosphorylation by GSK3β; of 76 tumors examined, approximately 13% had mutations within this domain, and 38% expressed β-catenin at high levels [44]. Similar to studies of ovarian and colorectal cancers, a relationship between nuclear β-catenin expression and stage, grade, and prognosis have been found in endometrial cancer [45], [46], [47], [48], [49]. Other studies have shown that whereas overall β-catenin levels correlate negatively with cancer grade, nuclear accumulation in cells at the invasive front of the tumor correlates positively with tumor stage, grade, and poor prognoses [50], [51], [52]. In one study, transgenic mice in which activated β-catenin was expressed specifically in the uterus exhibited high levels of β-catenin accumulation in nuclei, increased expression of c-myc and cyclin D1, increased glandular proliferation, and extensive hyperplasia – but no cancer within the first year of life [53]. Here we report on the role of the Wnt pathway and Lef1 specifically in endometrial cancer, introducing a role for overexpression of full-length Lef1 in the etiology of endometrial cancer, and for controlled expression of Lef1 in development of the endometrial gland.

In colorectal cancer, Lef1 expression is a prognostic factor related to survival, with high levels of Lef1 correlating with longer survival times [54]. In our study of endometrial cancers, we classified our samples according to distinct histologies, stages and grades. Given that the vast majority of our patients were still alive at the time of analysis, we were unable to perform survival analyses. However, we were able to consider Lef1 expression in relationship to advanced grade or aggressive histology. Our findings were consistent with those from colorectal cancers, in the sense that Lef1 expression was higher in cohorts with good prognosis. Lef1 mRNA was expressed at higher levels in endometrioid tumors than in papillary serous tumors and MMMT, and was expressed at higher levels in tumors of grades 1 and 2 than in those of grade 3 (Fig. 6). In grade 1 tumors, 95% of the malignant cells are contained within the glands lining the endometrium, whereas in grade 3 tumors, 50% of the malignant cells are contained within the glands. This finding supports a role for Lef1 overexpression in progression in the context of tumors originating from glandular elements (ie. grades 1 & 2) [33]. We also noted that Lef1 was expressed at higher levels within normal proliferative endometrium and in all endometrial tumors (regardless of histologic subtype) than in inactive endometrium. In an attempt to examine the role of Lef1 in cell-cycle regulation and metastasis, we examined the expression of two well-known Lef1 downstream targets, cyclin D1 and MMP7, in both normal endometrium and cancerous endometrium. Our discovery that the expression of both cyclin D1 and MMP7 was higher in endometrial tumors than in normal, non-cancerous endometrium is consistent with previous reports showing that both of these proteins are elevated in patients with endometrial cancer [12], [55].

There are several limitations to our study. We choose to analyze the level of Lef1 expression via RT-PCR instead of examining protein levels because we felt RT-PCR would be a more quantitative method to report degrees of Lef1 expression, as opposed to using Western blot to assay for protein expression. There are many factors that affect not only the production of Lef1 mRNA, but also the degradation, and as a result measurements of Lef1 mRNA may not accurately reflect the true amount of protein expression. Additionally our paper introduces the concept of Lef1 as a potential oncogene, and while we currently have data demonstrating the overexpression of Lef1 during development, the estrus cycle and in uterine cancer specimens from both mice and humans, we are lacking data that links this overexpression directly to hyperplasia and/or carcinoma formation. To confirm this idea, we currently have experiments ongoing in which Lef1 will be overexpressed in both *in vitro* and *in vivo* models, but this data is not yet mature. Currently we feel that our data supports the role of Lef1 as a biomarker of uterine growth (normal and cancerous) but whether Lef1 is an actually oncogene remains to be answered.

In conclusion, our study provides data in which Lef1 appears to be an integral regulator of normal endometrial gland development, whose inhibition disrupts gland formation. We have also shown developmental and cyclic variations of Lef1 protein expression within the mouse uterus. Lef1 is overexpressed in both mouse and human endometrial cancer specimens, and certain Lef1 downstream targets are activated through this mechanism, specifically cyclin D1 and MMP7. In cancerous specimens, Lef1 protein expression appears to be localized predominately within the glands. Additional studies are required to further elucidate the role of Lef1 in normal gland regulation and endometrial cancer formation.
